# Dynamic physiology and transcriptomics revealed the alleviation effect of melatonin on *Reaumuria trigyna* under continuous alkaline salt stress

**DOI:** 10.3389/fpls.2024.1486436

**Published:** 2025-01-21

**Authors:** Xuebo Li, Lei Wang, Heyi Wang, Rui Hao, Lunkai Gao, Hongbo Cui, Hai Wu, Xiaodong Wu, Tong Qiao, Weijie Bai, Liming Zhang

**Affiliations:** ^1^ Forestry College, Inner Mongolia Agricultural University, Hohhot, China; ^2^ Office of the People's Government of Haibowan District, District People’s Government of Haibowan District, Wuhai, Inner Mongolia Autonomous Region, China; ^3^ Office of the Bureau of Natural Resources, Natural Resources Bureau of Haibowan District, Wuhai, Inner Mongolia Autonomous Region, China; ^4^ Office of the Civil Affairs Bureau of Wuhai City, Civil Affairs Bureau, Wuhai, Inner Mongolia Autonomous Region, China

**Keywords:** salt-resistant plant, melatonin, dynamic physiology, transcriptomics, *Reaumuria trigyna*

## Abstract

**Introduction:**

*Reaumuria trigyna*, a pivotal salt-tolerant plant species in Central Asian salt desert ecosystems, has garnered significant attention due to its resilience under harsh environmental conditions. This study investigates the response mechanisms of melatonin on the dynamic physiology and transcriptomics of *Reaumuria trigyna*, a critical salt-tolerant plant species in Central Asian salt desert ecosystems. Despite significant progress in understanding plant salt tolerance, research on the positive effects of melatonin on *Reaumuria trigyna*, particularly its impact on seed germination and the underlying physiological and molecular mechanisms, remains limited.

**Methods:**

In this study, we evaluated the physiological responses of *Reaumuria trigyna* under continuous alkaline salt stress and examined the effect of melatonin on seed germination.

**Results:**

Our results demonstrate that melatonin at concentrations of 300μmol/L significantly enhances plant growth and promotes the accumulation of osmotic regulators. Notably, melatonin treatment increased the germination rate by 35.48% compared to the alkaline salt stress group, which exhibited a 52.15% lower germination rate than the untreated control. The key mechanism identified involves melatonin’s ability to increase antioxidant enzyme activity, reduce reactive oxygen species and hydrogen peroxide levels, and alter gene expression patterns.

**Discussion:**

Transcriptomic analysis revealed significant changes in gene expression, particularly in photosynthetic signal transduction, phytohormone signaling, MAPK signaling, and the peroxisome pathway, which are crucial for the plant’s response to alkaline salt stress. Our findings provide new insights into how melatonin affects plant growth, salt tolerance, seed germination, and gene expression in *Reaumuria trigyna* under continuous alkaline salt stress. These results address a significant gap in current scientific knowledge and offer valuable theoretical support and practical guidance for cultivating salt-resistant crops and the ecological restoration of salt-affected desert environments.

## 1 Introduction

Soil salinization has become an increasingly critical issue in recent years, driven by the combined effects of global climate change and human activity. The complexity and severity of this problem have intensified, presenting significant challenges to sustainable agricultural production and environmental management. The impact of saline-alkali soils on plant growth is multifaceted. First, the elevated concentration of soluble salts increases the osmotic pressure of the soil solution, limiting water uptake by plant roots and hindering seed germination. This leads to “physiological drought,” in which water loss from root cells causes wilting or even plant death, severely restricting growth and development (Wei et al., 2024). Second, the accumulation of salts in plant tissues disrupts cellular processes, damaging the protoplasm, interfering with protein synthesis, and impairing growth and development (Zhang et al., 2013). Furthermore, the direct toxicity of sodium bicarbonate and potassium carbonate to plant tissues can cause root damage and death, exacerbating the adverse effects of salinization.

Excess sodium ions in saline-alkali soils compete with plants for nutrient uptake, impairing the absorption of essential minerals such as potassium and phosphorus. This disruption of nutrient balance hinders plant growth and development (Tariq et al., 2023). Simultaneously, saline-alkali conditions can disturb the redox balance within plant cells, leading to the accumulation of key signaling molecules, such as reactive oxygen species (ROS) and calcium ions (Ca²^+^), which activate MAPKKK and MAPKK kinases (Zhang et al., 2024). This cascade induces oxidative damage to cellular structures, including chlorophyll, membranes, proteins, and nucleic acids. Salt-alkali stress also alters metabolite levels, such as sugars and phenylpropanoids, affects hormone receptor activity, and changes signal transduction molecules’ phosphorylation/dephosphorylation status, influencing downstream gene expression. These changes affect the sensitivity and specificity of secondary metabolite and hormone signaling pathways, indirectly impacting osmolyte content and antioxidant enzyme activity. For instance, salt stress may modulate the activity of enzymes like phenylalanine ammonia-lyase (PAL) and cinnamate-4-hydroxylase (C_4_H), which regulate phenylpropanoid biosynthesis (Shin et al., 2024; Song et al., 2022). Additionally, salt-alkali stress can inhibit the activity of critical enzymes in glycolysis and gluconeogenesis, such as hexokinase, phosphofructokinase, and pyruvate kinase, affecting carbohydrate metabolism and altering metabolic rates and pathways (Shinde et al., 2018). This disruption in metabolism may lead to the accumulation of metabolites such as lactate and glyoxylate, further influencing glycolysis and gluconeogenesis. Furthermore, under salt-alkali stress, abnormal interactions between AUX/IAA proteins and ARF transcription factors within the auxin (IAA) signaling pathway can modify the expression of downstream antioxidant enzyme genes, impacting plant growth and development (Bao et al., 2024). Plants activate antioxidant defense mechanisms to mitigate oxidative damage, including producing non-enzymatic antioxidants such as ascorbic acid (ASA) and glutathione (GR), which scavenge ROS and reduce oxidative stress. Plants generate osmolytes like proline (PRO) and total carbohydrates, which accumulate in cells to maintain osmotic balance and minimize the damage caused by salt-alkali stress (Gao et al., 2022).

*Reaumuria trigyna*, a salt-secreting and drought-tolerant shrub from the genus *Reaumuria* in the family Tamaricaceae (Chen, 1990), originated during the Tertiary period and is considered a relict species from the ancient Mediterranean, often referred to as a “living fossil.” Its current distribution is restricted to the eastern part of Alxa, Inner Mongolia, and the western region of Ordos, China (106°27’E to 111°28’E, 39°13’N to 40°52’N; elevations ranging from 1500 to 2100 m). As a typical halophyte, *Reaumuria trigyna* has garnered significant attention in botany, environmental science, and stress biology due to its exceptional salt tolerance and adaptive mechanisms. Scientific studies and clinical practice have also confirmed the plant’s efficacy in treating skin conditions such as eczema and dermatitis. Given the challenges posed by global climate change and land salinization, understanding the salt tolerance mechanisms and stress response strategies of *Reaumuria trigyna* is crucial for restoring and rehabilitating vegetation in saline-alkali soils. Recent research on *Reaumuria trigyna* has focused on its genomics and molecular biology (Zhang et al., 2023; Zhang et al., 2016), population genetic structure (Nui, 2024; Zhang & Wang, 2008), physiological and molecular responses to abiotic stresses (Zhang et al., 2023; Wang et al., 2022), salt tolerance mechanisms (Li et al., 2018; Du et al., 2019), and the impact of environmental factors on seed germination (Zhang et al., 2008). As a unique plant germplasm resource, *Reaumuria trigyna* plays a critical role in ecological restoration and vegetation recovery in desertified, arid, and semi-arid regions, owing to its distinctive ecological traits and adaptability. However, its high seedling mortality rate results in a low seed-to-seedling conversion rate, limiting subsequent population growth and contributing to its classification as an endangered species (Zhang et al., 2008). Consequently, the plant’s potential for ecological restoration remains underexplored. Direct seeding is a crucial method for environmental restoration, as it facilitates the rapid and complete restoration of vegetation structure and composition. Therefore, improving seed germination rates and the seed-to-seedling conversion rate of *Reaumuria trigyna* is essential.

Melatonin (N-acetyl-5-methoxytryptamine), an ancient biochemical compound (Lerner, 1959; Manchester et al., 2015), was first isolated from the pineal glands of cows (Lerner et al., 2002). Researchers initially identified melatonin as a critical regulator of animals’ biological clocks and circadian rhythms (Tan et al., 2009). Subsequent studies have shown that melatonin is present in animals and plants (Dubbels, 1995; Hattori, 1995), and its biological roles have expanded significantly. As a natural hormone with no known negative environmental impacts, melatonin has been widely used to promote seed germination, enhance plant growth, increase yields, and improve stress tolerance (Bartucca, 2022; Wu et al., 2021). For example, Yang et al. (2023) demonstrated that melatonin enhances blueberry fruit quality and its tolerance to Cd toxicity by regulating metabolites associated with ABC transporters, the TCA cycle, and flavonoid production. Duan et al. (2024) highlighted melatonin’s critical role in plant hormone synthesis, antioxidant enzyme production, and photosynthetic signal transduction. Yan et al. (2023) showed that melatonin improves wheat seedling salt tolerance by boosting antioxidant capacity and photosynthetic activity. Furthermore, several studies have indicated that melatonin increases plant tolerance to stress by modifying morphological and physiological traits (Sheikhalipour et al., 2023; Saqib et al., 2024; Fu et al., 2023). Regarding seed germination, Korkmaz et al. (2023) found that melatonin positively affects pepper seed germination and seedling growth under low temperature, high temperature, and water stress. Zeng et al. (2022) demonstrated that melatonin improves seed germination, seedling growth, and antioxidant defense in rice under submergence stress. These studies collectively show that melatonin enhances antioxidant capacity in plants by altering photosynthetic efficiency and physiological characteristics while stimulating the activity of various antioxidant enzymes, thereby mitigating stress-induced damage. Researchers have also explored the physiological response mechanisms of melatonin in seed germination and seedling growth by pretreating crops such as soybean sprouts (Wu et al., 2023), Zoysia japonica Steud (Dong et al., 2021), Stevia rebaudiana Bertoni (Simlat et al., 2018), and seeds of wheat, barley, oat, and soybean (Hernández-Ruiz et al., 2005; Wei et al., 2015). While much of the existing research on melatonin has focused on crops and herbaceous plants, few studies have investigated its effects on shrub growth and development. Moreover, no studies have examined the impact of melatonin on seed germination or seedling quality in *Reaumuria trigyna*. Seed germination is a critical stage in the plant life cycle, influencing subsequent growth and development. Learning the physiological responses and transcriptomic mechanisms underlying seed germination and their interconnections can enhance seed germination rates, improve seedling quality, optimize plant resource utilization, and promote sustainable plant development.

## 2 Materials and methods

### 2.1 Growth conditions and seedling treatments

The study was conducted at the College of Forestry, Inner Mongolia Agricultural University (111°41′E–111°37′E, 40°48′N–40°68′N) from March to June 2024. It comprised both a preliminary and a formal experiment, with seedlings collected from the wild in Wuhai City, Inner Mongolia (106°48′E–109°31′E, 38°45′N–40°45′N). The seeds were sterilized by immersion in a sodium hypochlorite solution for 15 minutes, followed by six rinses with distilled water and air drying. Previous studies have shown that melatonin concentrations ranging from 100 to 400 μmol/L can significantly activate the antioxidant mechanisms in various salt-tolerant shrubs and trees, including Salicornia fruticosa, apple trees, and soybeans (Zhao et al., 2023; Giménez et al., 2024; Xian et al., 2024). Wang (2023) found that a concentration of 50 mmol/L NaHCO_3_ significantly affected the growth of *Reaumuria trigyna*. We measured the pH and salt contents of nine major soil types in Wuhai City in May 2024. Our findings revealed that the pH of most soils fell within the range of 7.5 to 8.5 (**Table 1**). To make the experimental results more conducive to local ecological restoration, we adjusted the pH range for simulating salt-alkali stress to 8.2 to 8.6, aligning it more closely with actual conditions. Based on these findings, the concentrations of NaHCO_3_ and melatonin used in the present study were determined. The seeds were treated with 50 mmol/L NaHCO_3_ and soaked in various melatonin solutions for 24 hours, including the following treatments: CK (distilled water as a control), S (50 mmol/L NaHCO_3_), MT1 (100 μmol/L melatonin + 50 mmol/L NaHCO_3_), MT2 (300 μmol/L melatonin + 50 mmol/L NaHCO_3_), MT3 (500 μmol/L melatonin + 50 mmol/L NaHCO_3_), and MT4 (700 μmol/L melatonin + 5 NaHCO_3_). The treated seeds were then planted in plug trays (58 mm × 58 mm in diameter and 110 mm deep) filled with a white peat and fertilizer substrate and placed in an advanced greenhouse for cultivation. The greenhouse was maintained at 23 ± 3°C during the day and 15 ± 3°C at night, with relative humidity controlled between 60% and 70%. The greenhouse received natural light, with a 12-hour light-dark cycle. The substrate was kept moist through daily watering. NaHCO_3_ stress was applied every seven days to simulate continuous alkaline salt stress. The germination experiment was considered complete when no further germinations occurred after seven days. The formal experiment lasted 26 days, from May 24, 2024, to June 20, 2024. Each treatment group contained 100 seeds, with three replicates per treatment. *Reaumuria trigyna* seedlings showing uniform growth were randomly selected from each treatment group and stored in a -80°C freezer for physiological and biochemical analysis, as well as transcriptome sequencing.

**Table 1:** The pH and salt content of different soil types.

Soil type	Grey desert soil	Brown calcareous soil	Chestnut calcareous soil	Windblown dust soil	Meadow soil	Calcareous soil	Sandy soil	Coal cinder soil	Clay soil
PH	7.57 ± 0.03d	8.40 ± 0.02ab	8.56 ± 0.15a	8.35 ± 0.01abc	7.97 ± 0.02c	8.33 ± 0.04abc	3.77 ± 0.24e	8.14 ± 0.05bc	7.31 ± 0.56d
Salt content	4.25 ± 0.11c	3.52 ± 0.25d	1.76 ± 0.05e	6.88 ± 0.12a	4.71 ± 0.01b	1.31 ± 0.02f	0.00 ± 0.00g	4.72 ± 0.01b	1.72 ± 0.03e

Note: Data are presented as means ± SE (n = 3). This means that not sharing the same letter is significantly different at p ≤ 0.05 by DUNCAN’s LSD test.

### 2.2 Assessing physiological indexes

During the experiment, several parameters related to seed germination were calculated, including the number of seeds germinated, the number of dead seeds, germination peak time (the number of days from the start of the experiment to when the daily germination count reached its maximum), germination lag time (the time from the start of the test to the emergence of the first germinated seed), and germination duration (the total number of days from the start of the experiment to the germination of the last seed). Additionally, the following parameters were computed: average germination time (Equation 1), germination percentage (Equation 2), relative germination percentage (Equation 3), germination energy (Equation 4), germination index (Equation 5), vigor index (Equation 6), and seedling mortality rate (Equation 7). After the experiment, healthy seedlings with similar growth seedlings were selected on June 20, 2024. The seedlings’ surface soil was cleaned with distilled water, and surface moisture was blotted with filter paper. Measurements were then taken for stem length, primary root length, seedling height, leaf length, leaf width, and the number of leaves, using a vernier caliper with a precision of 0.001 inches. The calculation methods for these parameters are described in previous studies (Biju et al., 2017; Cao et al., 2019; Zhang et al., 2022).


(1) $$The\;average\;time\;of\;germination=\frac{\Sigma \left(n_{i}\times d_{i}\right)}{\Sigma n_{i}}$$

(2) $$GR=\frac{n}{N}\times 100\%$$

(3) $$RGR=\frac{GR\;of\;each\;group}{GR\;of\;CK}\times 100\%$$

(4) $$GP=\frac{N_{0}}{N}\times 100\%$$

(5) $$GI=\Sigma \left(n_{i}\times d_{i}\right)$$

(6) $$VI=GI\times (L_{1}+L_{2})$$

(7) $$DR=\frac{Number\;of\;dead\;seedlings}{N}\times 100\%$$

GR is the germination rate; RGR is the relative germination rate; GP is the germination potential; GI is the germination index; VI is the vitality index; DR is the number of seedling deaths; n is the number of seeds normally germinated during the experiment; N is the number of experimental seeds; n_0_ is the number of seeds that typically germinate when the number of daily germinated seeds reaches the peak; d_i_ is the number of days from the date of sowing, n_i_ is the number of normal germination on a corresponding day; l_1_ is the stem length, L_2_ is the main root length.

All physiological and biochemical measurements were conducted between June 21, 2024, and July 21, 2024. Root viability of the plant seedlings was assessed using the TTC (triphenyl tetrazolium chloride) method (Man et al., 2016). Seedling roots were cleaned and immersed in a TTC solution and phosphate buffer mixture in a dark, constant-temperature shaking incubator. After the reaction was stopped, the roots were crushed, and the extract was analyzed colorimetrically at 485 nm using a spectrophotometer to determine tetrazolium reduction intensity. MDA levels were measured using the thiobarbituric acid method, with absorbance readings taken at 450 nm, 532 nm, and 600 nm to calculate MDA content (Chen and Li, 2016). Photosynthetic pigments were extracted using the method developed by Lichtenthaler and Wellburn (1983), with 80% acetone as the solvent. The absorbance of the extract was measured at wavelengths of 470 nm, 663 nm, and 646 nm using a fluorescence spectrophotometer to determine the concentrations of chlorophyll a (Equation 8), chlorophyll b (Equation 9), and carotenoids (Equation 10). To prevent light-induced photosynthesis, we used blackout curtains to ensure that the experimental area remained completely dark throughout the experiment. Total carbohydrate content was determined using the anthrone colorimetric method (Li, 2000). The content of soluble protein was determined by the Coomassie brilliant blue method (Zhang and Qu, 2000). The proline content in leaf tissues was determined per 1 g of tissue fresh weight (FW) using the sulfosalicylic acid method (Zhang and Qu, 2000). The content of the O2- was determined by the hydroxylamine oxidation method. The content of ascorbic acid was determined by 2,6-dichlorophenol indophenol titration. The ultraviolet absorption method measured the hydrogen peroxide content and catalase activity (Aebi, 1984). Peroxidase activity was determined by guaiacol colorimetry. The activity of superoxide dismutase was determined by the nitrogen blue tetrazolium method. The ascorbate peroxidase activity was determined by spectrophotometry (Zou, 2000). Polyphenol oxidase activity, reduced glutathione content, and glutathione reductase activity were measured using kits. All the samples were collected in three independent biological replicates.


(8) $$C_{a}=12.21A_{663}-2.81A_{646}$$

(9) $$C_{b}=20.13A_{646}-5.03A_{663}$$

(10) $$C_{x\cdot c}=\frac{1000A_{470}-3.27C_{a}-104C_{b}}{229}$$

C_a_ represents the concentration of chlorophyll a; C_b_ represents the concentration of chlorophyll b; C_x·c_ represents the total concentration of carotenoids; A_663_, A_646_, and A_470_ represent the absorbance of the chloroplast pigment extract at wavelengths of 663nm, 646nm, and 470nm.

### 2.3 RNA extraction and transcriptomics analysis

To assess the effects of various treatments on leaf RNA, 0.2 g of leaf samples from each treatment group were collected and pooled to form a single biological replicate, with three replicates conducted on June 21, 2024. Total RNA was extracted using the R401 (Genepioneer Biotechnologies, Nanjing, China) and TSJ011-100 kits (Tsingke Biotechnology Co., Ltd., Beijing, China). The RNA concentration and purity were assessed using a NanoDrop 2000 spectrophotometer, and RNA integrity was verified by agarose gel electrophoresis (1% gel concentration, 180 V, 16-minute run time). The following criteria were met for library preparation and sequencing: no gel contamination, total RNA ≥ 1 ug, concentration ≥ 35 ng/uL, OD 260/280 ≥ 1.8, OD 260/230 ≥ 1.0, and sufficient total RNA for three library constructions. For library construction, eukaryotic mRNA was isolated using Oligo(dT) magnetic beads, randomly fragmented, and reverse transcribed into cDNA with six-base random primers. The cDNA was then processed by end repair, A-tailing, adapter ligation, fragment selection, and PCR amplification. Library quality was evaluated through preliminary quantification with Qubit 2.0, insert size determination using an Agilent 2100, and precise, effective concentration quantification (≥ 2 nM) via qPCR. Sequencing was performed on the Illumina NovaSeq X Plus platform with a read length of PE150. Transcriptome sequencing was outsourced to Genepioneer Biotechnologies (Nanjing, China).

### 2.4 Functional annotation of unigenes

Unigene sequences were aligned using BLAST software with several databases, including NR (non-redundant protein database), Swiss-Prot, GO (Gene Ontology), COG (Clusters of Orthologous Groups), KOG (eukaryotic Orthologous Groups), and KEGG (Kyoto Encyclopedia of Genes and Genomes). After predicting the amino acid sequences of the Unigenes, HMMER software was used to compare them with the Pfam database (Protein Families) for functional annotation.

### 2.5 qRT-PCR analysis

Six genes with differential expression were randomly selected for qRT-PCR analysis, performed by Genepioneer Biotechnologies (Nanjing, China). β-Actin was used as the internal reference gene, with primer sequences provided in **Supplementary Table 3**. The PCR protocol consisted of an initial denaturation at 95°C for 30 seconds, followed by 40 cycles of denaturation at 95°C for 5 seconds and annealing at 60°C for 20 seconds. Melting curve analysis was conducted from 60°C to 95°C, with fluorescence data collected at each one °C increment. Three biological replicates were included, and the error bars represent the Least Significant Difference (LSD). Relative expression levels of target genes were calculated using the 2^−ΔΔCT^ method.

### 2.6 Statistical calculation

Differential gene expression was analyzed using DESeq2 software, with a threshold of Log2FC > 1 and FDR< 0.05. The fold change (Log2FC) represents the ratio of expression levels between two samples or groups, while the p-value indicates the significance of differential expression. Plant transcription factors (TFs) were predicted using iTAK (V1.7a), which identifies TFs by applying hmmscan and predefined TF families from the database. Additionally, pathway identification and enrichment analysis were performed on the list of DEGs. A higher enrichment factor indicates a greater degree of enrichment, and the Q-value, ranging from 0 to 1, is the p-value adjusted for multiple hypothesis testing, with values closer to 0 indicating more significant enrichment. Physiological data were analyzed using SPSS Statistics 25, with one-way analysis of variance (ANOVA) to determine statistical differences. Results are presented as means of three biological replicates, with standard deviations (mean ± LSD). The LSD test (p< 0.05) was used to assess differences between treatment groups. Charts were generated using Microsoft Excel and Origin. Correlation and transcriptomic analyses were conducted using the “Chiplot” program to examine the effects of different melatonin concentrations on *Reaumuria trigyna*.

## 3 Results

### 3.1 Morpho-physiological parameters of melatonin to continuous alkaline salt stress in *Reaumuria trigyna*

**Figure 1:**
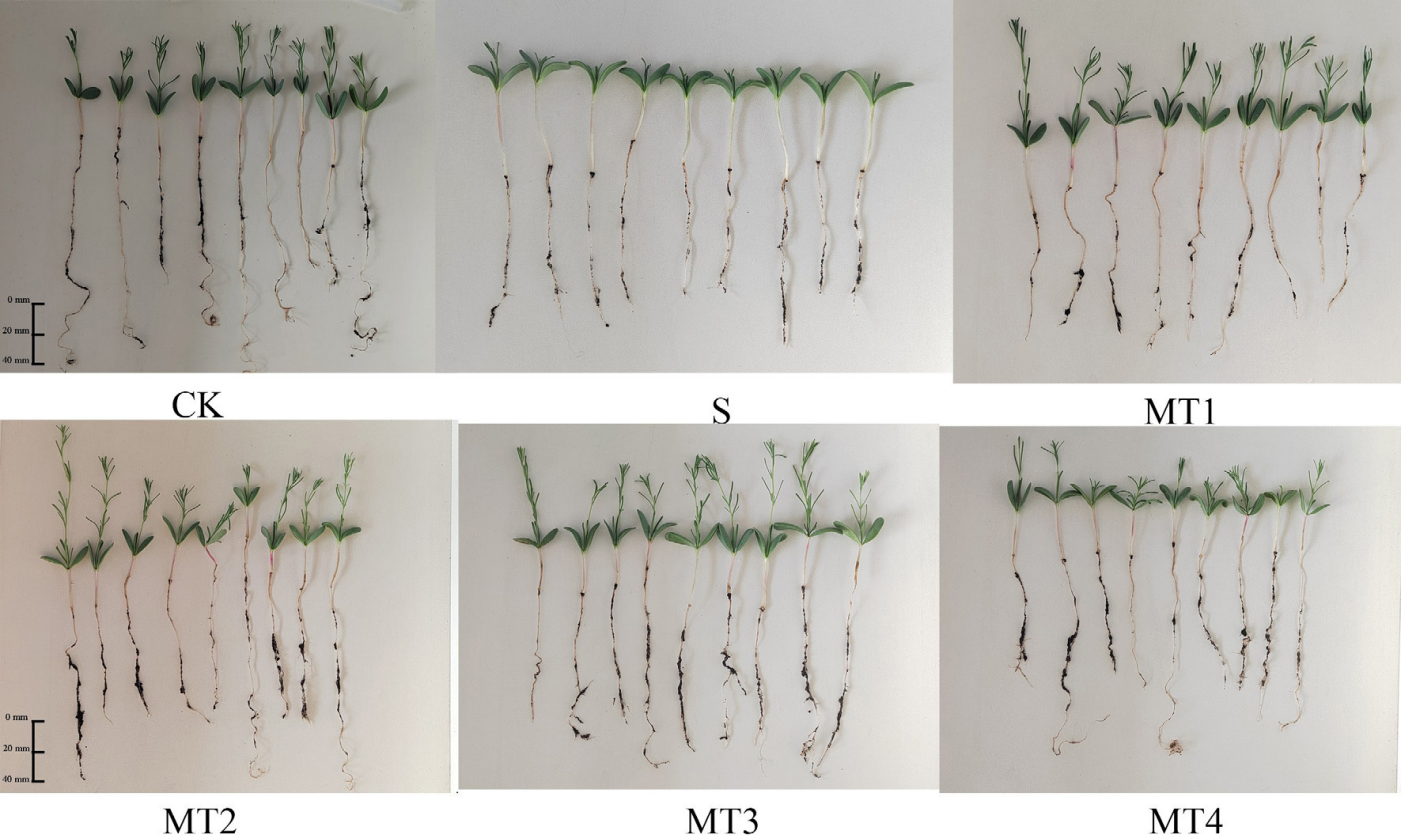
Effects of different melatonin concentrations on the morphology of *Reaumuria trigyna* seedlings.

**Figure 2:**
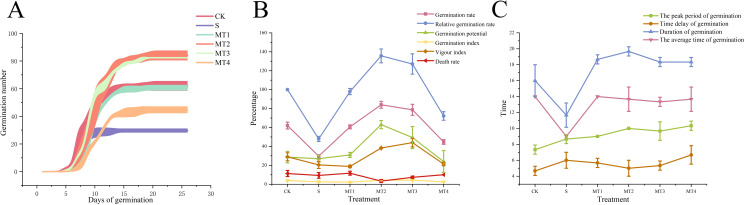
The effects of different concentrations of melatonin on the germination rate, germination characteristics, and germination time of *Reaumuria trigyna*. **(A)** represents the germination rate of *Reaumuria trigyna* seeds in different treatment groups; **(B)** represents the germination characteristics of *Reaumuria trigyna* seeds in different treatment groups; **(C)** represents the germination time of *Reaumuria trigyna* seeds in different treatment groups.

To investigate the physiological responses of *Reaumuria trigyna* to continuous alkaline salt stress under varying concentrations of melatonin, we subjected *Reaumuria trigyna* seeds to 50 mmol/L NaHCO₃ stress. And treated them with different doses of melatonin (100, 300, 500, and 700 µmol/L). The results (**Table 2**; **Figure 1**) revealed that seedlings exposed to alkaline salt stress alone (S) exhibited poor growth. Specifically, primary root length, seedling height, leaf length, leaf width, and leaf number were significantly reduced by 47.07%, 61.61%, 1.21%, 7.06%, and 67.86%, respectively, compared to the control group (CK), while stem length increased by 44.80%. In contrast, all morphological parameters improved substantially in the MT2 group. Seedling height increased by 39.49% compared to the CK and 263.34% relative to the S. The germination rate of the S was 52.15% lower than that of the CK. Additionally, compared to CK, the germination rates of MT2 and MT3 increased by 35.48% and 26.88% respectively. (**Figures 2A, B**). However, melatonin treatment (MT2) significantly enhanced the germination rate, relative germination rate, germination potential, germination index, and vigor index, while reducing the seedling death rate. Among these, the most notable improvements were observed in germination potential and seedling death rate, with germination potential increasing by 119.77% and 133.33% compared to the CK and S, respectively, and seedling death rate decreasing by 70.59% and 64.29%. Furthermore, as shown in **Figure 2C**, the S group markedly shortened the germination duration and reduced the average germination time.

**Table 2:** Effects of different melatonin concentrations on the growth of *Reaumuria trigyna* seedlings.

Treatment	Stem length(mm)	Root length(mm)	Seedling height (mm)	Leaf length(mm)	Leaf width(mm)	Leaves number
CK	24.19 ± 1.89b	142.13 ± 13.48a	43.10 ± 6.12c	19.05 ± 0.97b	9.31 ± 0.42b	19.00 ± 4.00ab
S	35.03 ± 3.76a	75.23 ± 1.80d	16.55 ± 2.97e	18.82 ± 0.48b	8.65 ± 0.88b	6.00 ± 1.00d
MT1	27.65 ± 1.03b	92.19 ± 5.60c	49.91 ± 4.93bc	18.85 ± 1.14b	8.71 ± 0.35b	16.00 ± 3.00bc
MT2	23.53 ± 1.29b	155.62 ± 15.25a	60.12 ± 6.67a	21.72 ± 1.53a	10.41 ± 0.45a	23.00 ± 2.00a
MT3	26.70 ± 3.03b	114.47 ± 3.43b	57.97 ± 2.14ab	21.00 ± 1.73ab	9.69 ± 0.76ab	22.00 ± 3.00a
MT4	27.77 ± 5.48b	111.68 ± 4.13b	32.77 ± 4.83d	19.13 ± 0.45b	8.89 ± 0.22b	13.00 ± 4.00c

Note: Data are presented as means ± SE (n = 3). This means that not sharing the same letter is significantly different at p ≤ 0.05 by DUNCAN’s LSD test.

**Figure 3:**
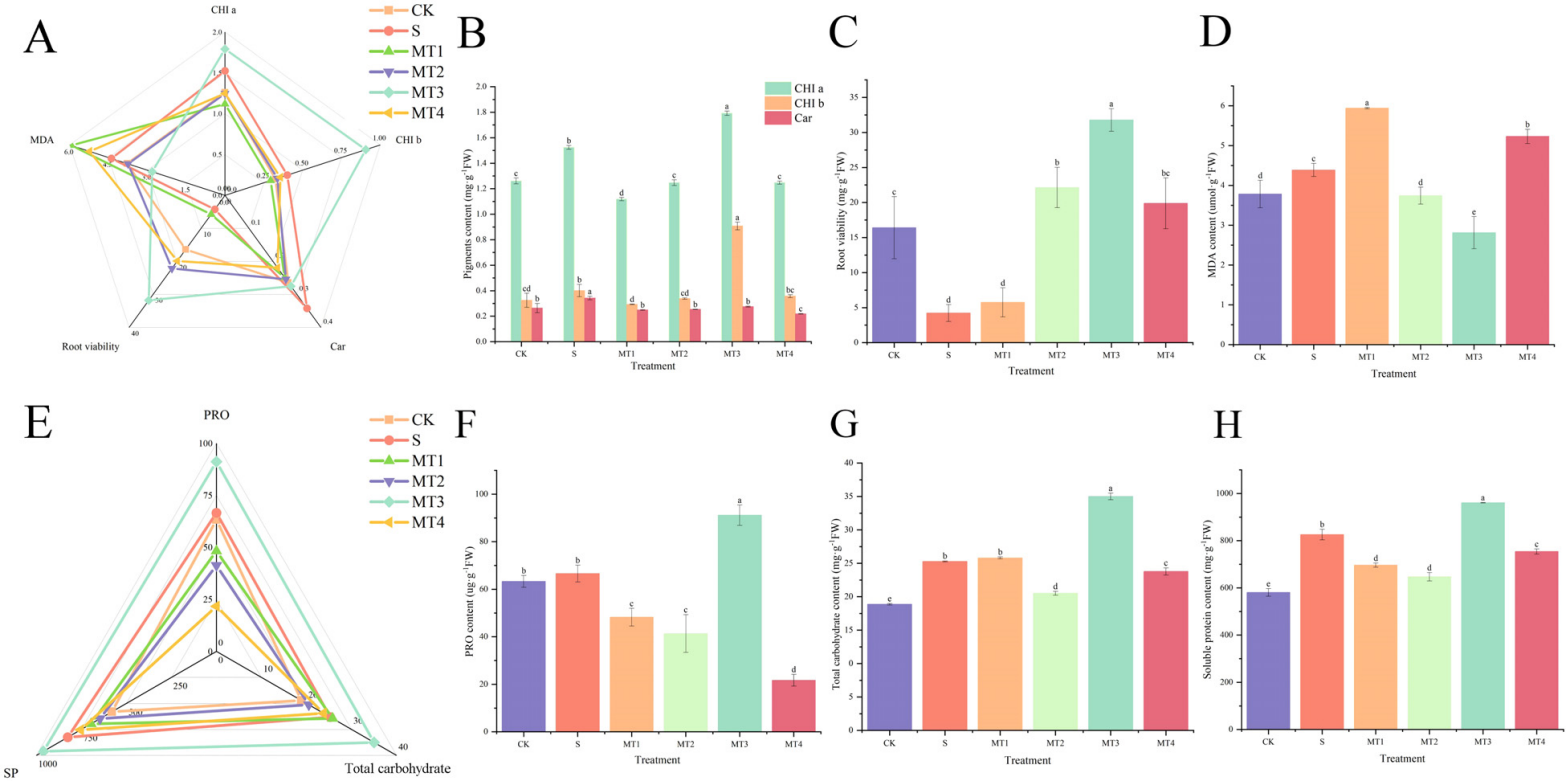
Effects of Various Melatonin Concentrations on Photosynthetic Pigments, Root Viability, MDA, and Osmoregulatory Compounds in *Reaumuria trigyna*. **(A)** is a radar chart illustrating the correlation between CHI a, CHI b, Car, MDA, and root viability, where different colored lines represent different treatment groups, a shorter distance between lines indicates a stronger correlation, and lines trending in the same direction indicate a significant positive correlation; **(B)** is the content of pigments; **(C)** is the MDA content; **(D)** is the root viability; **(E)** is a radar chart depicting the correlation between PRO, carbohydrates, and SP; **(F)** is the PRO content; **(G)** is the carbohydrate content; H is the SP content. Different lowercase letters indicate significant differences between treatments for the same species at the 0.05 level, the same as below.

**Figure 4:**
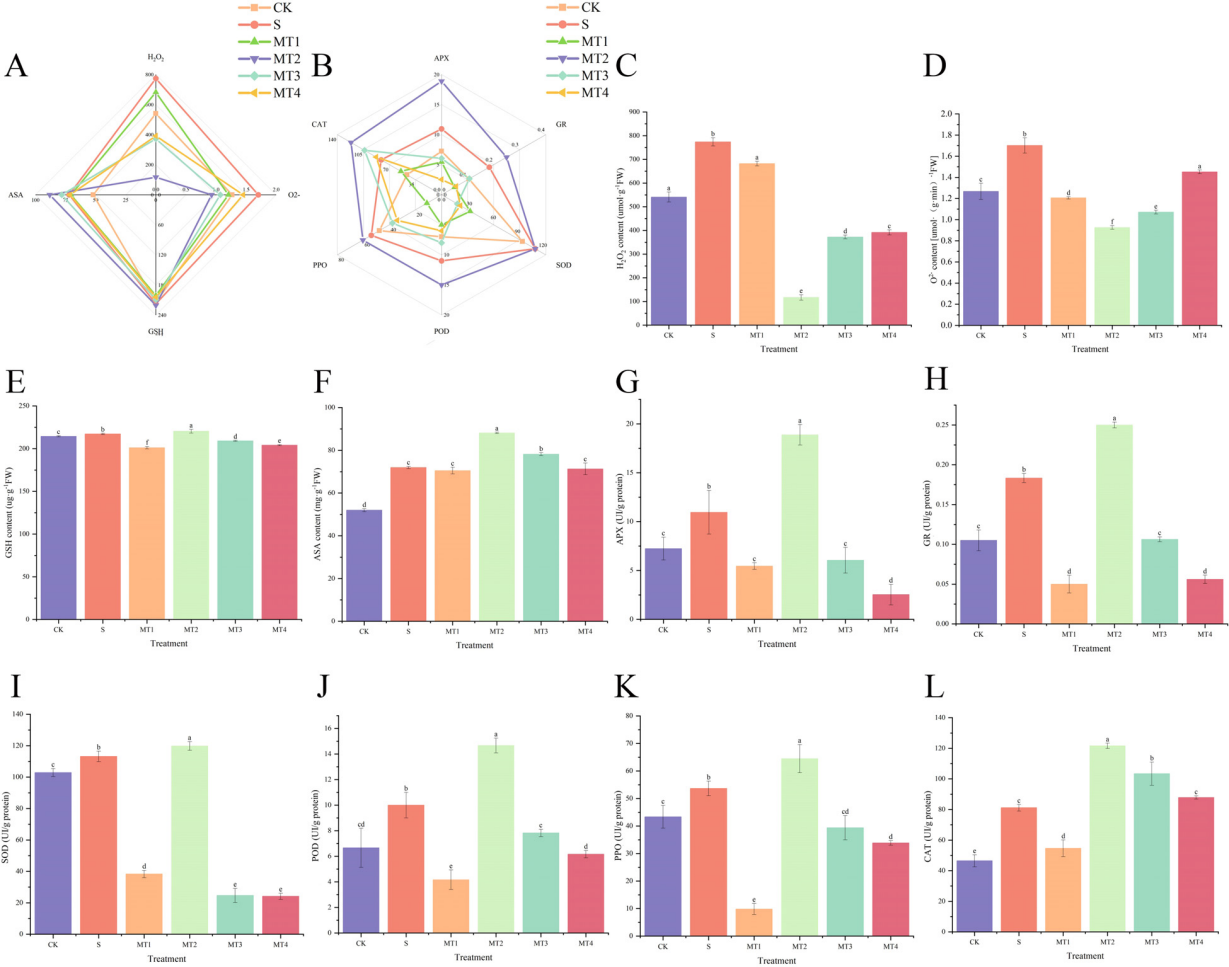
Effects of different concentrations of melatonin on antioxidant substances and antioxidant enzymes in *Reaumuria trigyna*. **(A)** is a radar chart illustrating the correlation between H_2_O_2_, O^2-^, ASA, and GSH; **(B)** is a radar chart illustrating the correlation between APX, GR, SOD, POD, PPO, and CAT, where different colored lines represent different treatment groups, a shorter distance between lines indicates a stronger correlation, and lines trending in the same direction indicate a significant positive correlation; **(C)** is the H_2_O_2_ content; **(D)** is the O^2-^ content; **(E)** is the GSH content; **(F)** is the ASA content; **(G)** is the activity of APX; **(H)** is the activity of GR; **(I)** is the activity of SOD; **(J)** is the activity of POD; **(K)** is the activity of PPO; **(L)** is the activity of CAT. Different lowercase letters indicate significant differences between treatments for the same species at the 0.05 level, the same applies hereinafter.

Our study demonstrated that a melatonin concentration of 500 µmol/L (MT3) was most effective in promoting the accumulation of photosynthetic pigments (**Figure 3B**) and osmotic regulators, as well as enhancing root activity (**Figure 3C**). At this concentration, malondialdehyde (MDA) content (**Figure 3D**) was significantly reduced, showing a decrease of 34.57% and 56.04% compared to the CK and S, respectively. The MT3 also lowered the levels of PRO (**Figure 3F**), total carbohydrates (**Figure 3G**), soluble proteins (SP) (**Figure 3H**), hydrogen peroxide (H_2_O_2_) (**Figure 4C**), and superoxide anion (O_2_⁻) content (**Figure 4D**). In contrast, the activities of key antioxidant enzymes, including GR (**Figure 4E**), ASA (**Figure 4F**), ascorbate peroxidase (APX) (**Figure 4G**), glutathione peroxidase (GSH) (**Figure 4H**), superoxide dismutase (SOD) (**Figure 4I**), peroxidase (POD) (**Figure 4J**), polyphenol oxidase (PPO) (**Figure 4K**), and catalase (CAT) (**Figure 4L**), were significantly enhanced. Notably, the activity of these antioxidant enzymes exhibited the most pronounced changes, increasing by 36.36%, 72.40%, 20.19%, 5.87%, 46.67%, and 49.91%, respectively, compared to the S. Surprisingly, the 100 µmol/L melatonin concentration (MT1) did not promote an increase in antioxidant enzyme activities in *Reaumuria trigyna* under continuous alkaline stress.

### 3.2 Correlation analysis of dynamic physiology of *Reaumuria trigyna*

**Figure 5:**
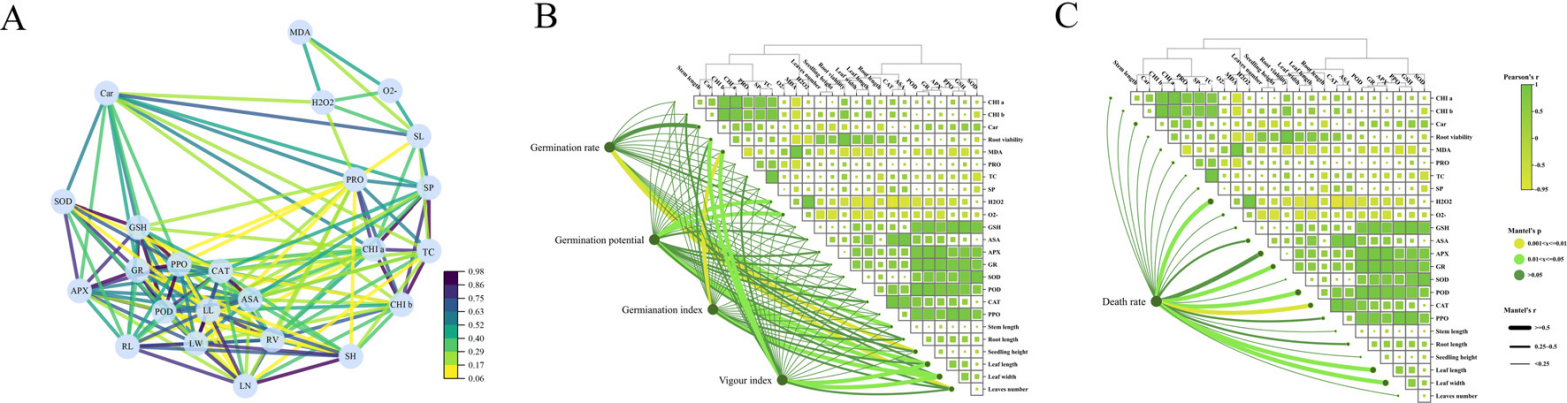
Correlation analysis of dynamic physiology of *Reaumuria trigyna* **(A)** is the neural network analysis diagram for the dynamic physiology of *Reaumuria trigyna*; **(B)** is the correlation heatmap between the dynamic physiology of *Reaumuria trigyna* and germination rate, germination vigor, germination index, as well as vigor index; **(C)** is the correlation heatmap between the dynamic physiology of *Reaumuria trigyna* and mortality rate. The correlation network diagram indicated a substantial association (p < 0.05) between the 23 assessed attributes across the six treatment groups. Each node represents a variable, and highly associated variables are grouped. Each path denotes one of the two variables it connects. POD is peroxidase; CAT is catalase; SOD is superoxide dismutase; PPO is polyphenol oxidase; H_2_O_2_ is hydrogen peroxide; O^2-^ is superoxide anion; GSH is glutathione; ASA is ascorbic acid; GR is glutathione reductase; APX is ascorbate peroxidase; MDA is malondialdehyde; PRO is proline; CHI a is chlorophyll a; CHI b is chlorophyll b; Car is carotenoid; RV is root activity; SP is soluble protein; SS is soluble sugar; RL is root length; SH is seedling height; LW is leaf width; LL is leaf length; SL is stem length; LN is the number of leaves. The color gradient in the correlation heat map symbolizes Pearson's correlation coefficient, while the line width denotes Mantel's r value.

As shown in **Figure 3A**, the root activity of *Reaumuria trigyna* under continuous alkaline salt stress, when treated with melatonin, exhibits a significant negative correlation with MDA levels, indicating that root activity increases as MDA content decreases. The trends for osmotic regulators (**Figure 3E**), antioxidant compounds (**Figure 4A**), and antioxidant enzyme activities (**Figure 4B**) remain consistent. The correlation network diagram in **Figure 5A** reveals that plant morphological traits (leaf length, leaf width, and leaf number) are strongly associated with antioxidant compounds and enzyme activities. The heatmaps in **Figures 5B, C** further illustrate that the germination indicators of *Reaumuria trigyna* seeds (germination rate, relative germination rate, germination index, and vigor index) exhibit weaker correlations with morphological traits but stronger associations with others, particularly antioxidant enzymes. Additionally, seedling mortality is correlated with ASA, H2O2, APX, CAT, SP, and morphological traits such as leaf length and width.

### 3.3 RNA extraction and transcriptome analysis

**Figure 6:**
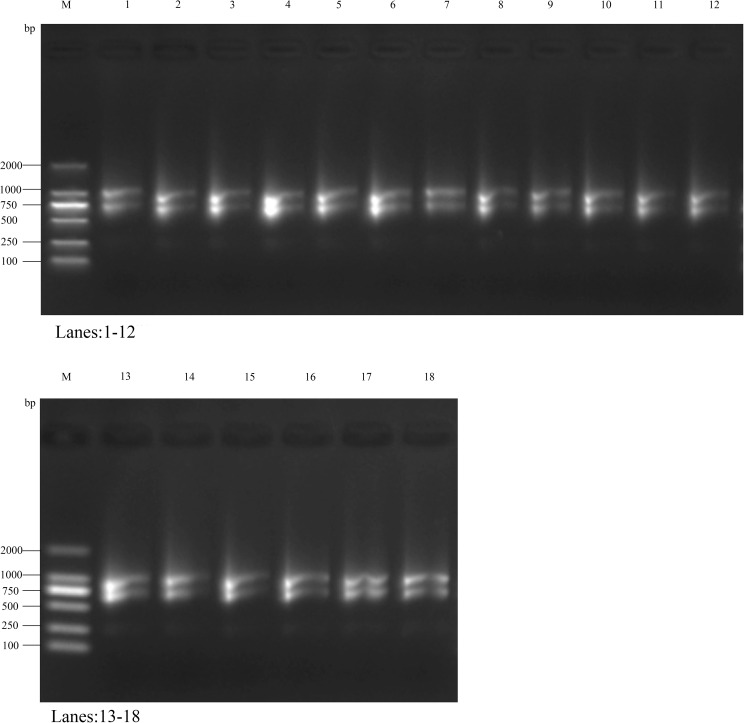
The results of agarose gel electrophoresis detection. Marker is DL2000 DNA Marker (100bp-2000bp).

The RNA purity of all samples was assessed by measuring the OD260/OD280 ratio using a UV spectrophotometer. The results showed that all samples’ OD260/OD280 ratio exceeded 2.0, indicating high RNA purity with minimal contamination from proteins or DNA. Additionally, the OD260/OD230 ratio ranged between 1.8 and 2.4, suggesting negligible residual salt contamination (**Supplementary Table 1**). RNA integrity was evaluated via agarose gel electrophoresis, which revealed distinct 28S and 18S rRNA bands in all samples. The 28S band intensity was approximately twice that of the 18S band, indicating good RNA integrity with no significant degradation (**Figure 6**).

We conducted a transcriptome analysis on the leaves of *Reaumuria trigyna* from different treatment groups. A total of 538.43 million raw reads were generated to investigate the molecular response of melatonin to alkaline salt stress. After filtering out low-quality reads, adapter contamination, and ambiguous sequences, we obtained 161.53 GB of clean data, with each sample providing at least 7.72 GB of data and a Q30 base percentage of 92.18% or higher (**Supplementary Table 2**). Sequence assembly was performed using Trinity, resulting in 573,530 transcripts and 249,784 unigenes. The N50 values for transcripts and unigenes were 1622 and 1148, respectively, indicating high assembly integrity. Of these, 60,274 unigenes were more significant than 1 kb in length. Functional annotation was carried out by comparing unigenes with the NR, Swiss-Prot, KEGG, COG, KOG, GO, and Pfam databases, yielding annotation results for 78,492 unigenes. Gene structure analysis, including SSR analysis, identified 36,721 SSR markers, and CDS prediction was also performed. Clean reads from each sample were aligned to the assembled reference genome, with alignment efficiency ranging from 79.60% to 80.72%, confirming the reliability of the sequencing results.

### 3.4 Functional annotation of unigenes

The functional annotation of unigenes revealed that the KEGG database had the highest annotation rate, with 95.59% (75,009), followed by NR with 92.44% (72,563), and KOG with 57.11% (44,828). The annotation rates for SwissProt, GO, Pfam, and COG were 50.54% (39,683), 45.67% (35,851), 33.37% (26,207), and 15.64% (12,275), respectively. Overall, 31.37% (78,492) of unigenes had successful annotations, while a significant proportion did not match any entries in public databases. This suggests that *Reaumuria trigyna* may exhibit high species-specificity, with many undiscovered genes, underscoring the importance of further molecular biology studies on this species.

**Figure 7:**
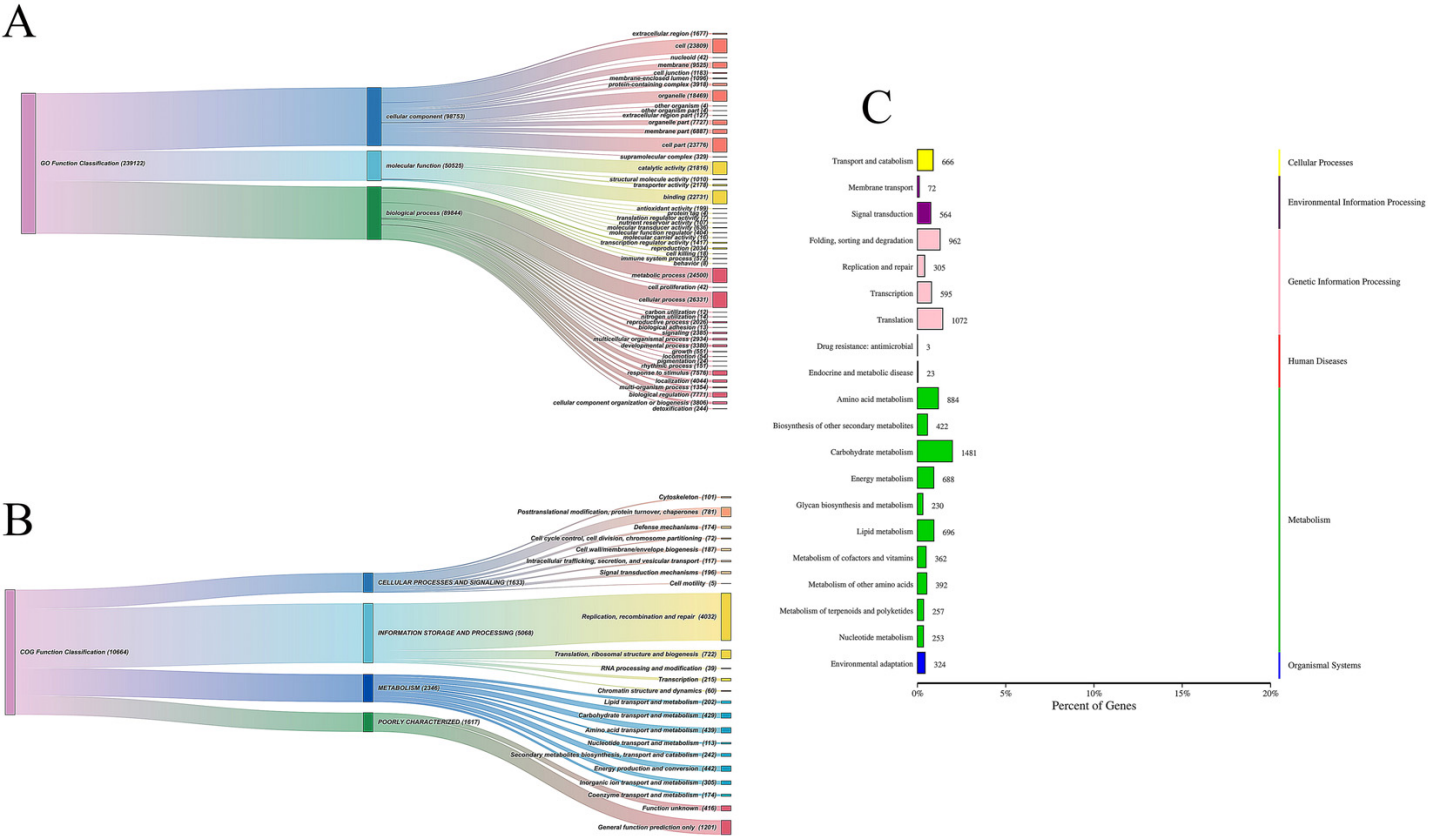
GO functional annotation **(A)**, COG functional annotation **(B)** and and KEGG pathway **(C)**.

Additionally, all KEGG-annotated unigenes were mapped to 134 pathways, which were primarily categorized into six major domains: cellular processes, environmental information processing, genetic information processing, human diseases, metabolism, and organismal systems (**Figure 7C**). Among these, the pathways related to carbohydrate, amino acid, and energy metabolism were the most prominent. The functional annotation results for GO and COG are shown in **Figures 7A, B**. GO annotations were classified into three broad categories: cellular component (98,753), molecular function (50,525), and biological process (89,844). The COG annotations were categorized into four main groups: cellular processes and signaling (1,633), information storage and processing (5,068), metabolism (2,346), and poorly characterized (1,617).

### 3.5 Analysis of DEGs

**Figure 8:**
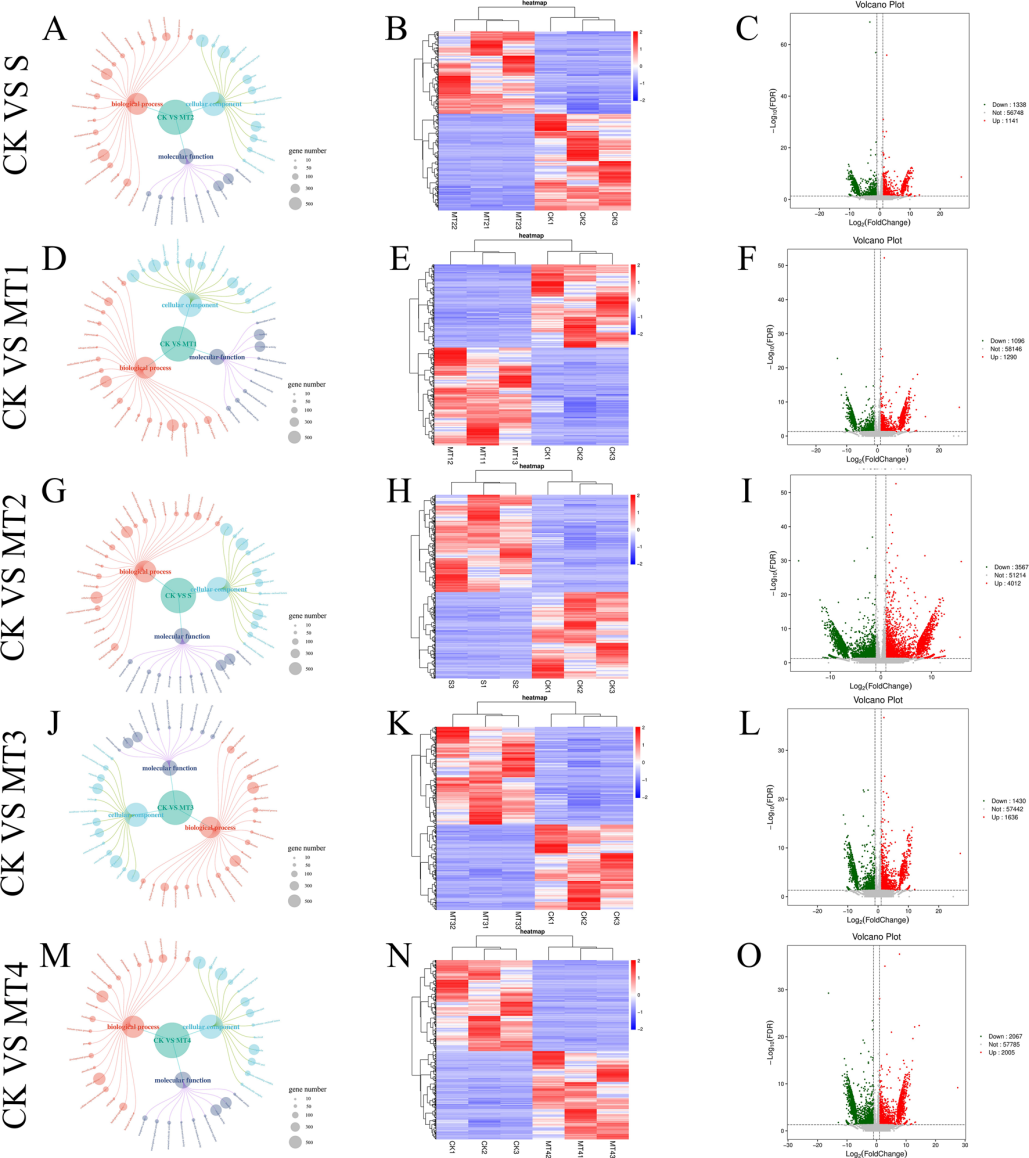
The GO function annotation correlation network diagram of DEGS **(A, D, G, J, M)**, the clustering diagram of DEGS **(B, E, H, K, N)**, the volcanic diagram of DEGS **(C, F, I, L, O)**. The size of the circles in the correlation network diagram represents the number of genes for each GO term. In the clustering diagram of DEGs, the horizontal axis represents the samples' sample names and clustering results, while the vertical axis represents the clustering results of DEGs and genes. Different columns in the diagram represent different samples, and different rows represent different genes. The color indicates the expression level of the gene in the sample. The diagram is plotted using the pheatmap function in R, and the row data is z-scale normalized using the scale function. In the volcano plot of DEGs, each point represents a gene, with the horizontal axis indicating the logarithmic value of the fold change in the expression level of a particular gene between two samples and the vertical axis indicating the negative logarithmic value of the FDR. A more considerable absolute value on the horizontal axis indicates a more significant difference in expression level between the two samples. A larger value on the vertical axis indicates more significant differential expression, and the screened differentially expressed genes are more reliable. Green points in the diagram represent downregulated differentially expressed genes, red points represent upregulated differentially expressed genes, and gray represents non-differentially expressed genes.

**Figure 9:**
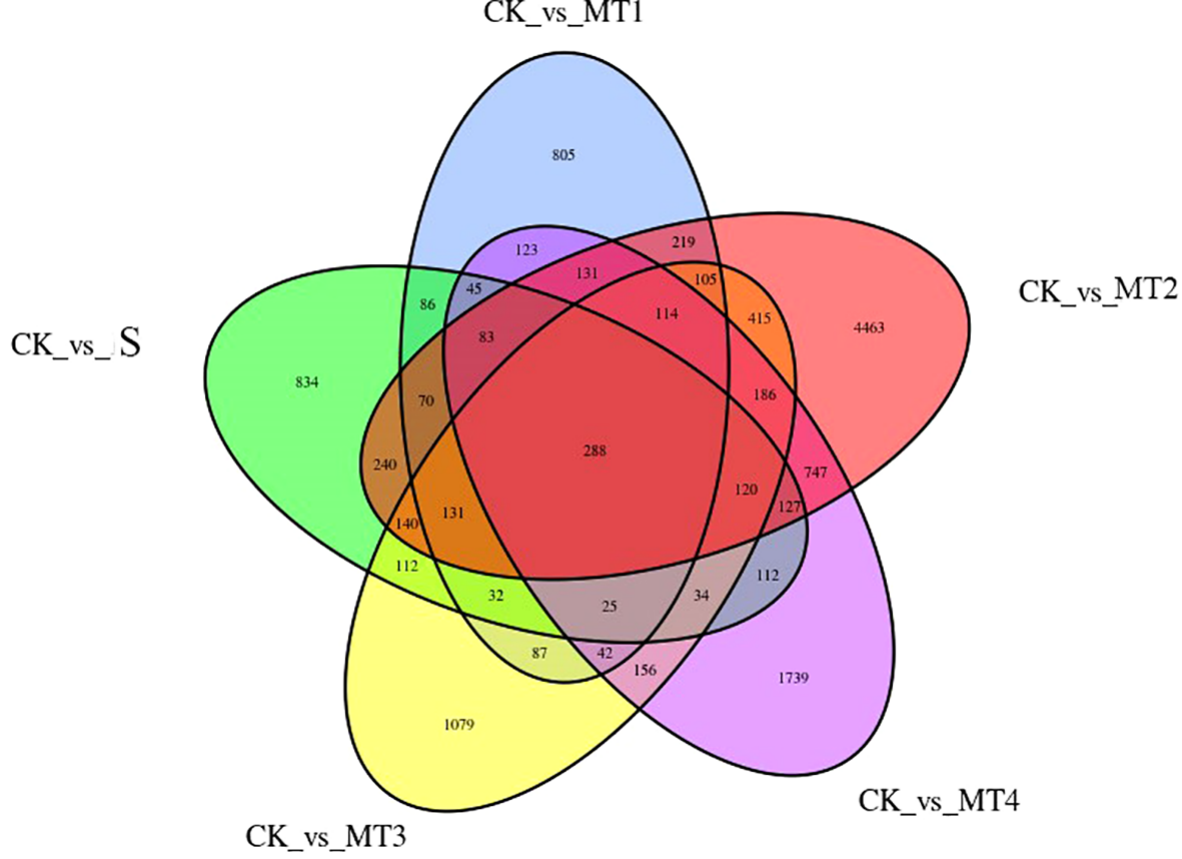
Venn diagram of DEGs Each circle represents a set of differential analysis combinations, and the number in the circle represents the number of differential genes in each combination.

Our study identified differentially expressed genes (DEGs) across five treatment groups: CK vs. S, CK vs. MT1, CK vs. MT2, CK vs. MT3, and CK vs. MT4. The total number of DEGs for each comparison was 2479, 2386, 7579, 3066, and 4072, respectively. The upregulated and downregulated genes in each group are presented in **Figures 8C, F, I, L, O**. Notably, in the CK vs. MT2, 4012 genes were upregulated, while 3567 genes were downregulated. To further assess the data, we performed hierarchical clustering analysis on the selected DEGs to group genes with similar or identical expression patterns (**Figures 8B, E, H, K, N**), confirming the experimental data’s reliability. Venn diagrams (**Figure 9**) were generated for each DEG comparison, illustrating the number of unique DEGs in each treatment group and the number of shared DEGs between groups. The results revealed 834, 805, 4463, 1079, and 1739 unique DEGs in the respective groups. The CK vs. MT2 and CK vs. MT4 groups also shared the highest number of DEGs, with 1964 common genes. 288 DEGs were shared across all six treatment groups, suggesting potential similarities in response or regulatory mechanisms at the transcriptomic level. Further research will focus on validating the functions of these shared DEGs and investigating the biological processes and regulatory mechanisms in which they may be involved.

### 3.6 Functional annotation and enrichment analysis of DEGs

In this experiment, the differentially expressed genes (DEGs) from the five comparative treatment groups were classified into at least 43 categories, with further subdivision into three main functional categories: Biological Process, Molecular Function, and Cellular Component. The GO terms with the highest number of differentially expressed genes were ‘cell part,’ ‘cellular process,’ and ‘metabolic process.’ In the GO analysis of the CK vs. S group, DEGs were significantly enriched in terms related to ‘cell’ and ‘catalytic activity,’ indicating that alkaline salt stress profoundly impacts plant cell structure and metabolic functions. In the CK vs. MT2 group, DEGs were enriched in terms such as ‘metabolic process,’ ‘catalytic activity,’ and ‘organelle’ (**Figures 8A, D, G, J, M**).

**Figure 10:**
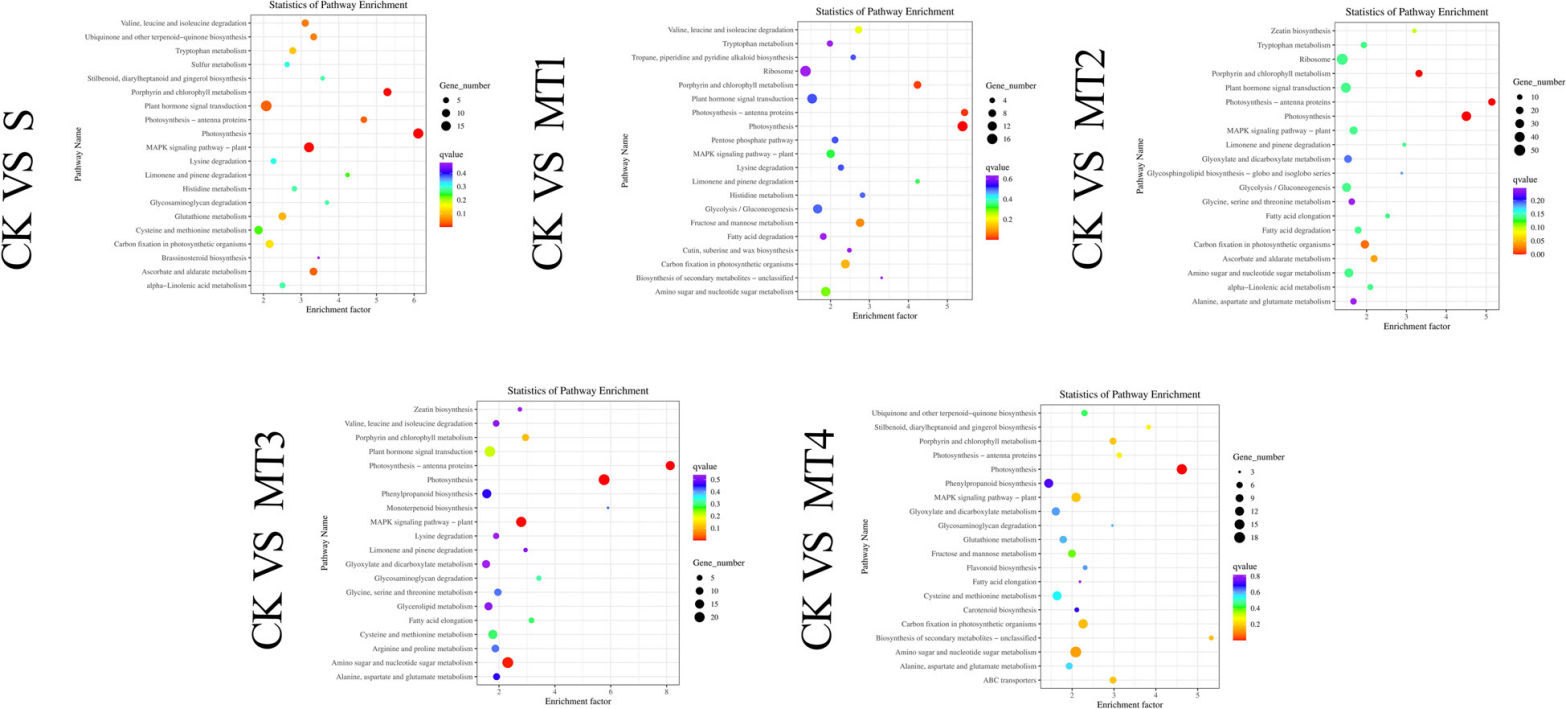
KEGG pathway enrichment scatter plot of DEGs Each row in the figure represents a KEGG pathway. The abscissa is the enrichment factor, indicating the ratio of the proportion of genes annotated to the pathway in the differential genes to the proportion of genes annotated to the pathway in all genes. The greater the enrichment factor, the more significant the enrichment level of differentially expressed genes in the pathway. The point's color represents Qvalue, and the size of the point represents the number of differentially expressed genes annotated in the pathway.

The KEGG functional annotation analysis of the DEGs revealed that 134 biological pathways contained DEGs across all comparison groups. In at least one comparison group (CK vs. MT1), 95 KEGG metabolic pathways were significantly enriched, with a false discovery rate (FDR)< 0.05 as the threshold. These pathways encompass various biological processes, including energy metabolism, substance synthesis and degradation, and signal transduction. Notably, in the CK vs. MT2 group, the most significantly enriched pathways included Plant Hormone Signal Transduction, Photosynthesis, Glycolysis/Gluconeogenesis, Amino Sugar and Nucleotide Sugar Metabolism, MAPK Signaling Pathway, Phenylpropanoid Biosynthesis, Peroxisome, Glutathione Metabolism, Arginine and Proline Metabolism, and Ascorbate and Aldarate Metabolism, among others. **Figure 10** presents the top 20 pathways with the lowest significance Q-values.

We compared the CK vs. S group with the CK vs. MT1, CK vs. MT2, CK vs. MT3, and CK vs. MT4 groups to further investigate the effects of melatonin on the metabolic pathways of the significantly enriched DEGs. We selected the most representative metabolic pathways from the CK vs. MT2 comparison for detailed analysis. We found that 39 DEGs were involved in the Plant Hormone Signal Transduction pathway. Compared to the CK vs. S group, 11 upregulated genes showed distinct responses, including the gene encoding serine/threonine-protein kinase SRK2 (EC: 2.7.11.1). Additionally, several genes were significantly upregulated, such as TRINITY_DN3653_c0_g1, TRINITY_DN6558_c0_g1, TRINITY_DN5305_c0_g1, and TRINITY_DN1580_c0_g1. These genes play various roles within this pathway, such as auxin-responsive protein IAA and the abscisic acid receptor PYR/PYL family, which are involved in plant growth and stress responses. Collectively, they form an essential network that regulates the biosynthesis of plant hormones, including IAA, cytokinins, gibberellins (GA), ABA, ethylene, brassinosteroids, JA, and salicylic acid. This network modulates the expression of antioxidant genes through signal transduction pathways, thereby regulating the activity of antioxidant enzymes (SOD and CAT) and enhancing the plant’s resistance to oxidative stress. Notably, among the DEGs enriched in Plant Hormone Signal Transduction, the gene encoding phytochrome-interacting factor 3 was upregulated, suggesting that melatonin application significantly influences plant light signal transduction. The MAPK (Mitogen-Activated Protein Kinase) signaling pathway involves 24 DEGs. It is illustrated as a cascade from MAPKKK (MAPK Kinase Kinase) to MAPK (MAPK Kinase), which regulates the expression and activity of antioxidant enzymes (SOD, CAT, POD, GR, APX) as well as the synthesis and accumulation of oxidative substances (ASA, GSH). This pathway is critical in plant growth, pathogen defense, and responses to environmental stresses.

**Figure 11:**
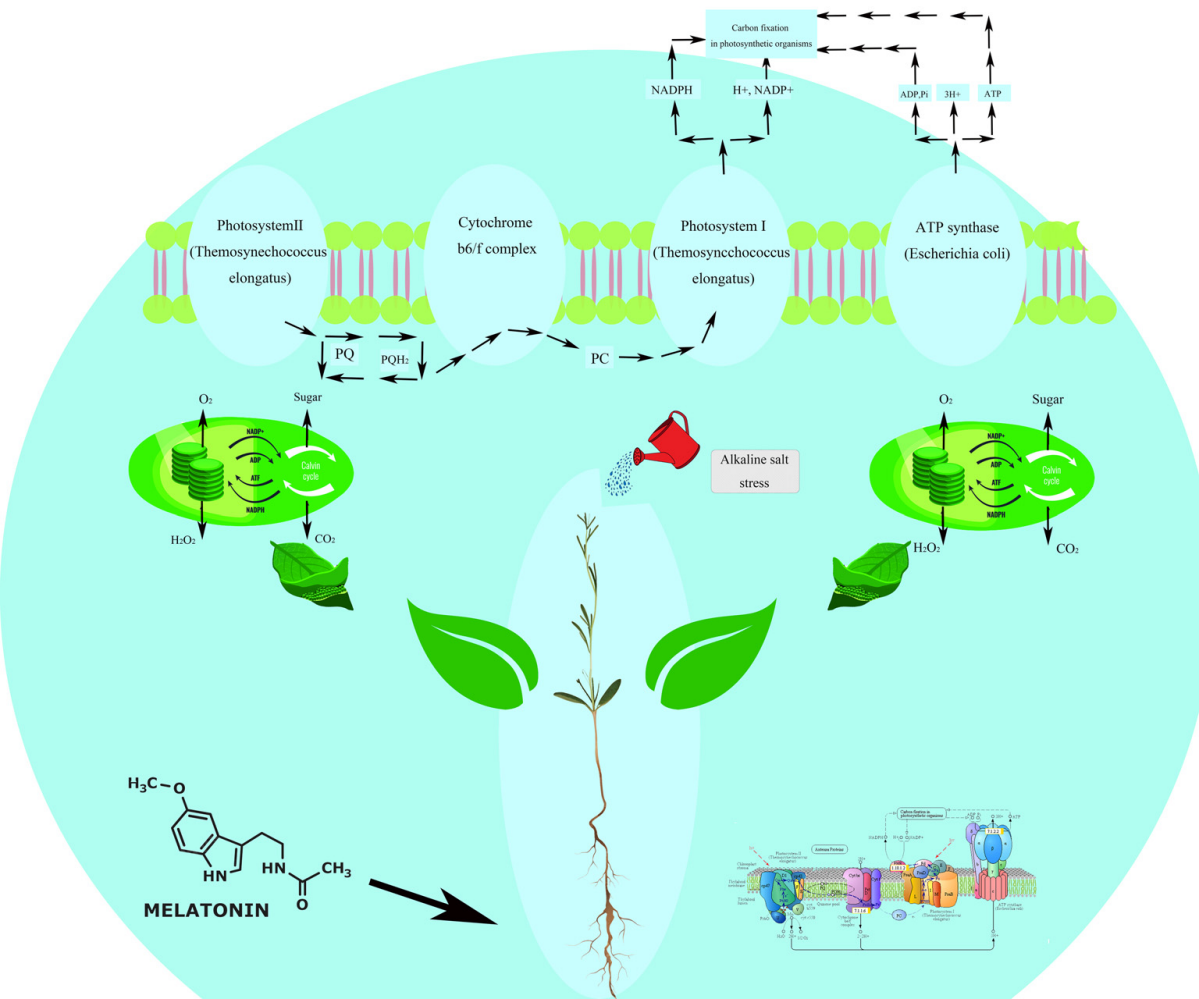
Photosynthetic mechanism of *Reaumuria trigyna*.

Additionally, many DEGs were activated in other metabolic pathways, including Peroxisome, Amino Sugar and Nucleotide Sugar Metabolism, Starch and Sucrose Metabolism, Photosynthesis (**Figure 11**), Glutathione Metabolism, Arginine and Proline Metabolism, Ascorbate and Alternate Metabolism, with 16, 31, 29, 36, 11, 14, and 17 DEGs, respectively. The Peroxisome pathway is complex. This pathway involves multiple key steps and enzymes/genes, including PTS receptors (PTS1, PTS2) involved in peroxisome biosynthesis and PEX genes (PEX1, PEX6, PEX10, etc.) that participate in peroxisome membrane formation and maintenance. PEX16 and PEX26 are crucial for receptor recycling. The figure also highlights critical enzymes involved in reactive oxygen metabolism, such as CAT and SOD. Specifically, enzymes like peroxidase [EC:1.11.1.7] (TRINITY_DN4720_c1_g2), Superoxide dismutase, Cu-Zn family [EC:1.15.1.1] (TRINITY_DN15126_c0_g1), (S)-2-Hydroxy-acid oxidase [EC:1.1.3.15] (TRINITY_DN4346_c0_g1), and Catalase [EC:1.11.1.6] (TRINITY_DN154513_c0_g1) were activated, with their encoding genes significantly upregulated. The significant enrichment of these pathways suggests that these metabolic processes are notably regulated under our experimental conditions.

### 3.7 Melatonin-induced transcription factors

**Figure 12:**
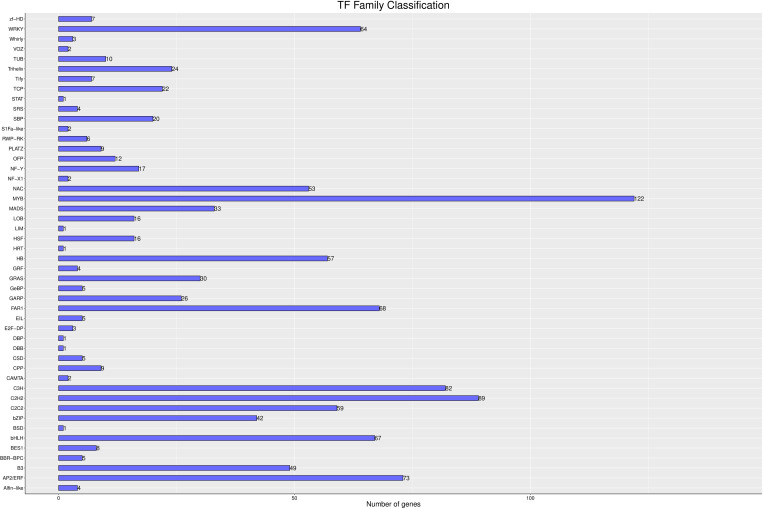
Transcription Factor Analysis The abscissa is different transcription factor families; the ordinate is the number of the transcription factor family.

Transcription factors (TFs) are crucial in the formation and maintenance of memory behaviors in both animals and plants. They bind to specific DNA sequences, known as cis-regulatory elements, in the promoter regions of target genes, thereby enabling precise regulation of gene expression. In this study, we investigated the impact of melatonin on the TF family. As shown in **Figure 12**, 1,149 genes were identified as TFs and classified into 48 distinct families. Notably, several critical transcription factor families, including WRKY (64 genes), NAC (53 genes), MYB (122 genes), HB (57 genes), FAR1 (68 genes), CH3 (82 genes), C2H2 (89 genes), BHLH (67 genes), B3 (49 genes), and AP2/ERF (73 genes), showed significant upregulation. These results suggest that melatonin may act as an important signaling molecule, modulating the expression levels of these transcription factors and playing a central role in the plant’s response to salt-alkali stress.

### 3.8 qRT-PCR validation assay

**Figure 13:**
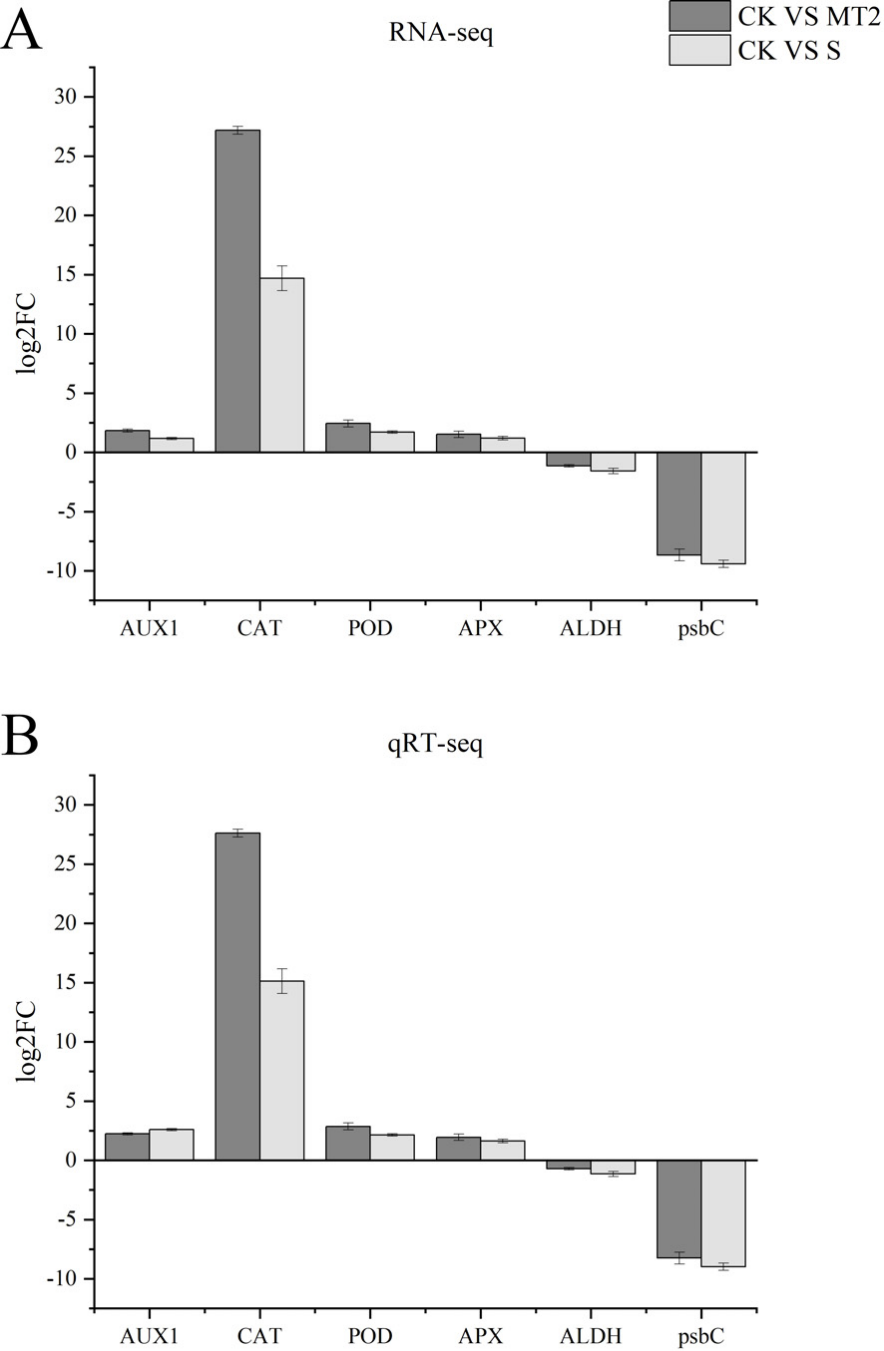
Verification of RNA-seq data by qRT-PCR. **(A)** is RNA-seq; **(B)** is qRT-seq.

The expression patterns of the six DEGs under both CK vs. S and CK vs. MT2 groups were identical. Four DEGs were significantly upregulated, including AUX1 (K13946), CAT (K03781), POD (K00430), and APX (K00434), while two DEGs were downregulated: ALDH7A1 (K14085) and psbC (K02705). The expression trends of these six DEGs under both CK vs. S and CK vs. MT2 groups were consistent with the RNA-seq results (**Figure 13**), confirming the high reliability of the transcriptome data.

## 4 Discussion

Alkaline salt stress affects plants at multiple levels, including physiological, biochemical, molecular, and metabolic processes. Our experiments revealed that exposure of *Reaumuria trigyna* to a 50 mmol/L NaHCO_3_ solution significantly reduced seed germination and seedling growth, underscoring the detrimental effects of alkaline salt stress. Notably, treatment with an optimal melatonin concentration (300 μmol/L) substantially improved germination rate, vigor index, and morphological parameters while reducing seedling mortality. These results highlight melatonin’s potential role in enhancing plant survival under stress conditions (Arnao and Hernández-Ruiz, 2020; Manzoor et al., 2023; Zhang et al., 2014). These findings have important ecological implications since *Reaumuria trigyna* is a key ultra-xerophyte for ecological restoration in arid regions. Most importantly, melatonin has positively affected plant physiological traits and enzyme activity across various species. For instance, it has enhanced growth and development in Solanaceae plants under abiotic stress (Ali et al., 2023). Ubaidillah et al. (2024) reported that melatonin promotes rice growth under salt stress, reduces ROS and MDA levels, and enhances its antioxidant capacity. Overall, melatonin acts as a free radical scavenger, efficiently neutralizing ROS such as O^2-^ and H_2_O_2_ in numerous species, including edible plants and legumes (Tan, 1993; Manchester et al., 2000; Yolanda et al., 2015). Through both its direct antioxidant action and its ability to activate antioxidant enzyme systems, melatonin significantly boosts plant stress resistance. These mechanisms, which mitigate oxidative stress and maintain membrane integrity, are consistent with those observed in other species under saline-alkaline stress, highlighting the broad potential of melatonin to enhance plant resilience.

We compared the effects of melatonin under saline-alkali stress with those of other protective compounds, such as EBR, MeJA, and ABA, previously studied in *Reaumuria trigyna* under similar stress conditions (Khan et al., 2023). Despite the use of different compounds, some experimental results overlap with our findings. For example, several studies have reported that protective compounds can significantly enhance plant antioxidant enzyme activity, thereby mitigating oxidative damage caused by saline-alkali stress. Additionally, other studies have shown that these compounds can regulate plant osmotic pressure, further improving plant adaptation to high saline-alkali environments (Wang, 2019; Xing, 2021). In conclusion, our study confirms melatonin’s efficacy as a protective compound under saline-alkali stress but also reveals potential common response patterns between melatonin and other protective compounds. These findings offer valuable insights into plant adaptation mechanisms to alkaline stress.

We can identify the molecular processes underlying plant responses to abiotic stress by comparing the DEGs across different groups. This approach helps to screen genes for stress resistance and provides valuable genetic resources for developing stress-tolerant crop varieties. Functional annotation and enrichment analysis revealed that, compared to the CK vs. S group, the four signaling pathways—Plant Hormone Signal Transduction, MAPK Signaling, Peroxisome, and Photosynthesis—were significantly more enriched in the CK vs. MT2 group, with a notable increase in the number of associated DEGs. Therefore, we propose that melatonin positively affects plants under alkaline salt stress primarily through these four pathways. Melatonin regulates the synthesis and signaling of auxin, a crucial plant hormone. Tryptophan, the precursor of auxin (IAA), undergoes complex biochemical transformations to form IAA, which is then transported into cells via the AUX1 protein. Upon binding with the TIR1 receptor, IAA activates signal transduction pathways involving AUX/IAA proteins and ARF transcription factors, ultimately regulating the expression of downstream auxin-responsive genes and antioxidant enzyme genes. This regulation influences antioxidant enzyme activity, as well as cell expansion and growth, contributing to stress tolerance. These findings align with the research of Li et al., 2024, who demonstrated that melatonin enhances salt and alkali tolerance in poplar by modulating plant hormone signal transduction pathways. Our study further supports the conclusion that melatonin protects plants from abiotic stress by promoting plant hormone synthesis.

The MAPK signaling pathway is another critical target through which melatonin enhances plant stress tolerance. This pathway recognizes pathogen-associated molecular patterns (PAMPs). It activates defense responses, exemplified by the FLS2 receptor-mediated activation of the MEKK1/MKK4/5-MPK3/6 cascade. This cascade induces the expression of defense-related genes, promoting the synthesis of disease-resistant compounds, such as PAD3. The MAPK signaling system also involves various biological processes, including wound response and ROS homeostasis via the MKK3/MPK8 pathway. Previous research has shown that specific MAPK members can directly or indirectly influence key components of the ABA signaling pathway, which is essential for plant stress responses (Liang et al., 2024; Khan et al., 2022). Melatonin may modulate the activity of these MAPK members, thereby regulating ABA signaling and enhancing plant tolerance to salt stress (Ipsita et al., 2024). Sheikh et al. (2024) demonstrated that melatonin interacts with MAPK proteins to regulate downstream gene expression. Our study found that melatonin treatment activated MAPK pathways, resulting in the upregulation of genes encoding antioxidant enzymes. These findings suggest that melatonin may regulate antioxidant gene expression through MAPK-mediated signaling. As a potent antioxidant, melatonin enhances plant resilience under saline-alkali stress by boosting peroxisome function, which is crucial for fatty acid β-oxidation, hydrogen peroxide metabolism, and ROS scavenging, thereby protecting cells from oxidative damage. Our results further elucidate the beneficial role of melatonin in activating the MAPK and Peroxisome pathways, thereby enhancing plant adaptation to salt-alkali stress.

Photosynthesis is plants’ most critical metabolic pathway (**Figure 11**), as it underpins essential physiological processes and plays a vital role in plant stress resistance. Melatonin, a hormone found in plants and animals, exerts a broad range of physiological functions, significantly enhancing the contribution of photosynthesis to plant stress tolerance through several mechanisms. Firstly, as a potent antioxidant, melatonin scavenges excess ROS, reducing oxidative damage to photosynthetic structures and maintaining the stability and efficiency of the photosynthetic system, even under stressful conditions. Secondly, melatonin promotes chlorophyll synthesis, increasing chlorophyll content and enabling plants to capture more light energy. This enhances photosynthetic efficiency and slows chlorophyll degradation under stress, extending leaves’ photosynthetic lifespan (Fan et al., 2024). Finally, melatonin induces the expression of photosynthesis-related genes, whose protein products play critical roles in stress responses, including protecting photosynthetic structures from damage and regulating the antioxidant system (Zhao et al., 2024). In summary, melatonin modulates photosynthesis through multiple pathways, including its antioxidant activity and regulation of chlorophyll synthesis, collectively enhancing plant survival and reproductive capacity in dynamic and challenging environments.

The WRKY family of transcription factors plays a crucial role in regulating the expression of antioxidant enzymes such as SOD, CAT, and POD. By binding to specific cis-elements in the promoters of these genes, WRKY proteins can either activate or repress their expression, modulating the plant’s antioxidant capacity. For example, under salt stress, the expression of WRKY genes in *Oryza sativa* L. increases significantly, with the SOD gene family showing strong upregulation. This suggests that WRKY transcription factors contribute to rice’s adaptability to salt stress by regulating antioxidant enzyme genes like SOD (Huang et al., 2021). In our study, we observed that the upregulation of WRKY genes was accompanied by increased activities of SOD, CAT, and POD, leading to a reduction in ROS levels. This indicates that WRKY transcription factors will likely enhance the plant’s antioxidant defense system in response to saline-alkali stress. Similarly, overexpression of MYB transcription factors, such as GmMYB68 and GmMYB3a, significantly affects the physiological indices of transgenic soybeans under saline-alkali stress, including changes in soluble sugar content, PRO, and antioxidant enzyme activity. This suggests that GmMYB68 and GmMYB3a play critical roles in soybean’s response to saline-alkali stress by regulating antioxidant gene expression (Duan et al., 2024). Our study found that MYB genes were upregulated in response to saline-alkali stress, potentially contributing to the increased production of antioxidants that scavenge ROS. Furthermore, the FAR1 family, a group of transcription factors derived from transposase enzymes, is critical for initiating the phytochrome A (phyA) signaling pathway. Although the specific roles of the FAR1 family in plants remain underexplored, its origin and association with phytochrome A signaling suggest that it may play an essential role in the light signal response, as well as in regulating plant growth and development (Tang et al., 2012).

Our findings reveal patterns of gene expression during seed germination, with AUX1, CAT, POD, and APX genes exhibiting an upregulation trend (**Figure 13**). AUX1, functioning as a key auxin influx carrier, plays a pivotal role in modulating seed germination rates. Notably, the upregulation of AUX1 observed in response to melatonin treatment suggests a mechanism by which seeds maintain high germination rates under stressful conditions. This is achieved through enhanced auxin levels and distribution within the radicle, ultimately activating downstream CYCD-mediated cell division. In parallel, the significant increase in antioxidant enzyme activity driven by the upregulation of CAT, POD, and APX genes underscores their critical role in bolstering the seed’s antioxidant defenses and stress tolerance. These genes synergistically promote normal seed germination and enhance stress resilience by regulating auxin distribution, scavenging reactive oxygen species, and participating in cell wall modifications. Conversely, the downregulation of ALDH7A1 and psbC genes observed in our study warrants further investigation. Preliminary hypotheses suggest that the downregulation of ALDH7A1 may be intricately linked to the regulation of aldehyde metabolism under stress conditions, while the suppression of psbC may impair photosystem II function, thereby adversely affecting photosynthesis efficiency and seed germination. These observations not only contribute to our understanding of the complex molecular mechanisms underlying seed germination and stress tolerance but also highlight potential genetic targets for the enhancement of crop stress resistance through advanced genetic engineering approaches. Future studies are needed to elucidate the precise roles of these genes in seed biology and their potential applications in crop improvement.

In summary, our study investigates the previously unexplored effects of melatonin on *Reaumuria trigyna*, providing novel insights into its potential mechanisms of action. Through transcriptome analysis, we identified significant changes in gene expression, particularly in pathways related to stress responses, antioxidant defense, and osmotic regulation. These findings highlight melatonin’s role in modulating key physiological processes. Furthermore, we discovered that melatonin regulates several critical metabolic pathways, including the plant hormone signal transduction pathway, MAPK signaling pathway, peroxisome pathway, and photosynthesis, enhancing our understanding of plant functions and their contribution to stress tolerance. These results expand the current knowledge of melatonin’s role in plant biology and open new avenues for future research on its potential applications in enhancing plant resilience.

## 5 Conclusion

This is the first study to elucidate melatonin response mechanisms on the dynamic physiology and transcriptomics of *Reaumuria trigyna*, providing new insights into ecological restoration and stress resistance studies with xerohalophytes. To summarize, salt-alkali stress greatly retards plant growth and development, reduces photosynthesis, and promotes the formation of ROS. However, melatonin administration can considerably reduce the damage produced by salt-alkali stress on plants. Melatonin maintains intracellular osmotic balance and enhances the antioxidant defense system by synthesizing osmoregulatory substances such as PRO, SP, and total carbohydrate. This increases the activity of antioxidant substances and enzymes, effectively scavenging ROS and protecting cells from oxidative damage. Melatonin stimulates the expression of several genes associated with stress resistance, including ion transporter genes, antioxidant-related genes, and signal transduction-related genes. Essential genes in specific metabolic pathways, such as the MAPK signal transduction pathway, were also significantly differentially expressed. Finally, metabolic pathway research revealed that melatonin administration influences several essential metabolic pathways, including plant hormone synthesis, photosynthesis, sugar metabolism, and peroxisome biosynthesis. This adds to our understanding of melatonin’s relief mechanisms. Notably, we discovered that melatonin can promote the expression of several transcription factors, which may operate as essential regulatory nodes and play an important part in the process by which melatonin alleviates salt stress. These integrated response mechanisms interact at numerous levels, assisting *Reaumuria trigyna* in maintaining life activities during stress and offering opportunities for survival and reproduction in salt-alkali stress settings. This reveals its significant adaptation and resistance tactics to salt-alkali stress, which mitigate or negate the deleterious effects of high-salt settings. These findings support melatonin’s beneficial impact in mitigating plant damage caused by abiotic stress.

## Data Availability

The datasets analyzed during the current study are available in the NCBI repository, National Center of Biotechnology Information under BioProject accession number PRJNA1163768. https://www.ncbi.nlm.nih.gov/bioproject/PRJNA1163768/.
