# Supplementary material for: Dynamic physiology and transcriptomics revealed the alleviation effect of melatonin on Reaumuria trigyna under continuous alkaline salt stress
Source: Front Plant Sci. 2025 Jan 21;15:1486436. doi: 10.3389/fpls.2024.1486436 (PMC11790669; doi:10.3389/fpls.2024.1486436)
Supplement: Supplementary file 1 [file Table1.docx]

Supplementary Table 1 Results of RNA extraction

| Sample | Concentration  (ng/μl) | Volume  (μl) | Total Quantity  (ug) | OD  260/280 | OD  260/230 |
| --- | --- | --- | --- | --- | --- |
| CK1 | 225.88 | 40 | 9.04 | 2.13 | 2.30 |
| CK2 | 199.33 | 40 | 7.97 | 2.14 | 2.41 |
| CK3 | 244.02 | 40 | 9.76 | 2.13 | 2.32 |
| S1 | 370.67 | 40 | 14.83 | 2.14 | 2.30 |
| S2 | 287.02 | 40 | 11.48 | 2.14 | 2.30 |
| S3 | 369.42 | 40 | 14.78 | 2.14 | 2.35 |
| MT11 | 224.67 | 40 | 8.99 | 2.16 | 1.93 |
| MT12 | 218.94 | 40 | 8.76 | 2.15 | 2.40 |
| MT13 | 211.53 | 40 | 8.46 | 2.14 | 2.15 |
| MT21 | 252.68 | 40 | 10.11 | 2.14 | 1.87 |
| MT22 | 211.06 | 40 | 8.44 | 2.14 | 2.40 |
| MT23 | 239.43 | 40 | 9.58 | 2.14 | 2.36 |
| MT31 | 306.38 | 40 | 12.26 | 2.14 | 2.35 |
| MT32 | 235.57 | 40 | 9.42 | 2.14 | 2.01 |
| MT33 | 210.44 | 40 | 8.42 | 2.15 | 1.94 |
| MT41 | 230.74 | 40 | 9.23 | 2.13 | 2.39 |
| MT42 | 279.01 | 40 | 11.16 | 2.13 | 2.34 |
| MT43 | 281.75 | 40 | 11.27 | 2.14 | 2.35 |

Supplementary Table 2 Illumina NovaSeq X Plus was used to sequence the data of different treatment groups of *Reaumuria trigyna*.

| Sample | ReadSum | BaseSum | GC(%) | N(%) | Q20(%) | CycleQ20(%) | Q30(%) |
| --- | --- | --- | --- | --- | --- | --- | --- |
| CK1 | 26125404 | 7837621200 | 44.86 | 0 | 97.23 | 100 | 92.18 |
| CK2 | 28828984 | 8648695200 | 44.18 | 0 | 97.24 | 100 | 92.24 |
| CK3 | 36637881 | 10991364300 | 44.30 | 0 | 97.29 | 100 | 92.50 |
| S1 | 30740328 | 9222098400 | 44.24 | 0 | 97.44 | 100 | 92.85 |
| S2 | 31131668 | 9339500400 | 44.55 | 0 | 97.32 | 100 | 92.54 |
| S3 | 26742442 | 8022732600 | 44.44 | 0 | 97.24 | 100 | 92.21 |
| MT11 | 32921451 | 9876435300 | 44.32 | 0 | 97.39 | 100 | 92.73 |
| MT12 | 31271585 | 9381475500 | 44.44 | 0 | 97.29 | 100 | 92.42 |
| MT13 | 31680066 | 9504019800 | 44.23 | 0 | 97.39 | 100 | 92.72 |
| MT21 | 32239399 | 9671819700 | 44.25 | 0 | 97.44 | 100 | 92.9 |
| MT22 | 30007775 | 9002332500 | 44.34 | 0 | 97.44 | 100 | 92.87 |
| MT23 | 28310999 | 8493299700 | 44.5 | 0 | 97.26 | 100 | 92.27 |
| MT31 | 31120749 | 9336224700 | 44.31 | 0 | 97.43 | 100 | 92.82 |
| MT32 | 25746629 | 7723988700 | 44.37 | 0 | 97.31 | 100 | 92.52 |
| MT33 | 29682943 | 8904882900 | 44.45 | 0 | 97.35 | 100 | 92.57 |
| MT41 | 27851516 | 8355454800 | 44.55 | 0 | 97.34 | 100 | 92.52 |
| MT42 | 27946100 | 8383830000 | 44.34 | 0 | 97.42 | 100 | 92.80 |
| MT43 | 29444355 | 8833306500 | 44.34 | 0 | 97.48 | 100 | 92.99 |

ReadSum： The total number of pair-end Reads in the Clean Data ( one pair of reads is counted at both ends of read1 and read2 here ); BaseSum: Clean Data total base number; GC ( % ): Clean Data GC content, that is, the percentage of G and C bases in Clean Data to the total bases; N( % ): N base content in Clean Data; Q20 ( % ): the percentage of bases with Clean Data mass value greater than or equal to 20; Q30 ( % ): the percentage of bases with Clean Data mass value greater than or equal to 30. Each treatment group was repeated three times.

Supplementary Table 3 Primers used for the qRT-PCR validation

| Gene ID | Forward primer（5′ to 3′） | Reverse primer（5′ to 3′） |
| --- | --- | --- |
| β-Actin | GGAATCCACGAGACCACCTACA | GATTGATCCTCCGATCCAGACA |
| AUX1 | GCTTAGTGTTTCGCTTCATCG | TCCTTCCAACGCAATACAGA |
| CAT | TAGAATTGTGCTCACCACGG | CTGGACCTCTTTCTCCGACA |
| POD | GGTGCTCATACAATCGGCTT | CCTGAGCGAATCTGGGATTA |
| APX | TTGATCTGAAGAGGGCCAAG | TACGGAGAAGAAATGCCTGC |
| ALDH | AATCAAACAAGTTTGCGATGC | TTGATCACCAACCCTTCCTC |
| psbC | ACAATTAACTAAATAAAATCTTCCCCT | CACGAGTTCAAACCCTTCGT |
